# Inequalities in maternity care and newborn outcomes: one-year surveillance of births in vulnerable slum communities in Mumbai

**DOI:** 10.1186/1475-9276-8-21

**Published:** 2009-06-05

**Authors:** Neena Shah More, Ujwala Bapat, Sushmita Das, Sarah Barnett, Anthony Costello, Armida Fernandez, David Osrin

**Affiliations:** 1 Society for Nutrition, Education and Health Action (SNEHA), Urban Health Centre, Chota Sion Hospital, 60 Feet Road, Shahunagar, Dharavi, Mumbai 400017, Maharashtra, India; 2 UCL Centre for International Health and Development, Institute of Child Health, 30 Guilford St, London WC1N 1EH, UK

## Abstract

**Background:**

Aggregate urban health statistics mask inequalities. We described maternity care in vulnerable slum communities in Mumbai, and examined differences in care and outcomes between more and less deprived groups.

**Methods:**

We collected information through a birth surveillance system covering a population of over 280 000 in 48 vulnerable slum localities. Resident women identified births in their own localities and mothers and families were interviewed at 6 weeks after delivery. We analysed data on 5687 births over one year to September 2006. Socioeconomic status was classified using quartiles of standardized asset scores.

**Results:**

Women in higher socioeconomic quartile groups were less likely to have married and conceived in their teens (Odds ratio 0.74, 95% confidence interval 0.69–0.79, and 0.82, 0.78–0.87, respectively). There was a socioeconomic gradient away from public sector maternity care with increasing socioeconomic status (0.75, 0.70–0.79 for antenatal care and 0.66, 0.61–0.71 for institutional delivery). Women in the least poor group were five times less likely to deliver at home (0.17, 0.10–0.27) as women in the poorest group and about four times less likely to deliver in the public sector (0.27, 0.21–0.35). Rising socioeconomic status was associated with a lower prevalence of low birth weight (0.91, 0.85–0.97). Stillbirth rates did not vary, but neonatal mortality rates fell non-significantly as socioeconomic status increased (0.88, 0.71–1.08).

**Conclusion:**

Analyses of this type have usually been applied across the population spectrum from richest to poorest, and we were struck by the regularly stepped picture of inequalities within the urban poor, a group that might inadvertently be considered relatively homogeneous. The poorest slum residents are more dependent upon public sector health care, but the regular progression towards the private sector raises questions about its quality and regulation. It also underlines the need for healthcare provision strategies to take account of both sectors.

## Background

The world is urbanizing in both absolute and relative terms. About half of humanity now lives in towns or cities (3 billion people, the same number as lived in the world in 1960) [1]. By 2015, 21 of 23 global megacities (>10 million inhabitants) will be in developing countries [2]. Mumbai is one of them and illustrates the fact that, in urbanizing fastest, Asia has the largest proportion of people living in slums [3]. Because slums are difficult to define clearly, a United Nations expert group recommends a provisional operational definition based on multiple domains: inadequate access to safe water, sanitation and other infrastructure, poor structural quality of housing, overcrowding, and insecure residential status [4].

Urban life is both good for you and bad for you. Although residents of cities historically paid an urban penalty, urban mortality in developing countries has been lower than rural mortality for about 50 years [5]. Concentration of population – and wealth – makes for better services, and water supply, sanitation, schools and healthcare are usually better than national averages [6]. The trade-off is complex: infant mortality rates fall with increasing urbanisation, but may rise with the proportion of the population living in slums [2]. Where disadvantages arise, they particularly affect women and children, in whom associations between slum conditions and illness have been described [7-17]. It has been suggested that infant and under-five mortality rates for the poorest 40% of the urban population are as high as in rural areas [18]. More than a quarter of the world's neonatal deaths occur in India [19]. Recent estimates for Mumbai suggest an infant mortality rate of 30 and a neonatal mortality rate of 25 per thousand live births, respectively [20]. Although antenatal care and institutional delivery are common (around 90%), we estimate the maternal mortality rate for women living in slums at about 200 per 100 000 live births (unpublished figures from surveillance). Women living in urban slums are more vulnerable because of (among others) earlier and less stable sexual relations, shorter breastfeeding duration, environmental risks, increased household labour and the need to purchase fuel, water and food, and a lack of the social networks found in rural areas [15].

The degree to which aggregate urban health statistics mask intra-urban inequalities is unclear. Most commentators call for an examination of health and healthcare indicators at a less generalised level, particularly in terms of slum populations and intra-urban disparities [18,21-24]. We are concerned with improving the health of women and children in urban slums, but our knowledge of maternal and newborn care-seeking and outcomes in urban communities is limited at a disaggregated level [9,10,12,25-29]. India's 2001 Census identified 1959 slum settlements in Mumbai, home to 54% of the city's 16.4 million people, but covering only 6% of its land area [30]. As part of the City Initiative for Newborn Health [31], we have access to a community-based maternity surveillance system, covering a population of about 300 000 in vulnerable slum areas. We used information collected for all births occurring over one year to examine the heterogeneity of maternity care practices and outcomes. We know that, at a gross population level, it is possible to demonstrate inequalities in health care and health between groups based on quantiles of socioeconomic status. We wondered whether these patterns would be replicated within a sample who all lived in slum conditions and would all be described as poor; whether an overall pattern of inequality would be repeated within a segment of the distribution.

## Methods

### Study location and population

The Municipal Corporation of Greater Mumbai provides services across 24 urban wards in three zones. The Corporation's Department of Public Health administers tertiary medical colleges, specialist hospitals, peripheral general hospitals, maternity homes, dispensaries, and health posts. Within a broad range of programmes, these provide preventive, promotive and curative services for mothers and children. Corporation hospitals contribute about 11 900 of Mumbai's estimated 40 000 hospital beds [32]. The rest fall under the private sector [33], which encompasses a range of services from large hospitals, through small nursing homes with a few beds, to single-handed general practitioners.

We collected information on maternity and newborn care for births in 48 vulnerable slum clusters, using the surveillance system set up for a cluster randomised controlled trial [34]. The system was run for a year to provide baseline information. The sampling frame included vulnerable areas of slums in six municipal wards (F North, G North, H East, K West, M East, P North). These were selected purposively for accessibility and to reflect a range of infant mortality rates according to Municipal Corporation estimates. We identified vulnerable slum areas in two stages. First, we discussed vulnerability criteria with a range of key informants (women's group leaders, health workers, ration shopkeepers, community leaders, political leaders, members of community-based organisations, non-government organisation workers, community health volunteers). The discussions yielded a list of 117 potential clusters. In the second phase, we visited each area to classify vulnerability systematically. We defined vulnerability in terms of higher proportions of social risk indicators (unemployment, groups in difficult circumstances, substandard housing), environmental indicators (open drainage, informal water supply, informal electricity supply), and health service utilisation indicators (infrequent interaction with community health volunteers, home deliveries). Inclusion was limited by four considerations: (1) levels of migration that would make follow-up impossible (construction workgroups, transit camps, pavement dwellings), (2) a strong likelihood of slum demolition in the next three years, (3) the need to avoid contiguous clusters as far as possible in order to minimise contamination across the subsequent trial, and (4) a minimum cluster size of 1000 households.

92 candidate vulnerable slum clusters were identified across the 6 wards, after which sampling was stratified by ward: eight clusters were selected randomly from each, giving a total of 48. For study purposes, each cluster contained 1000–1500 households; some clusters included whole slum areas, while others corresponded with geographical sub-areas. The 48 sample clusters were mapped and their boundaries clarified. A vital registration system was set up to identify births, stillbirths, neonatal deaths and maternal deaths. The system was adapted from successful ones in rural Nepal [35], and the Indian states of Jharkhand and Orissa [36].

Data for the study originated from a trial approved by the Municipal Corporation of Greater Mumbai, the Independent Ethics Committee for Research on Human Subjects (Mumbai committee, reference IEC/06/31), and the ethics committee of the Institute of Child Health and Great Ormond Street Hospital for Children.

### Procedures

The study was headed by a project coordinator (NSM) and data collection activities were managed by two project officers (UB and SD), each responsible for three wards. Vital events were identified by 99 locally resident women, generally 2 per cluster, each covering an average 600 households. Preference in recruiting community identifiers was given to married women with some stature in the community and remuneration was based on verified events. Births and deaths were communicated to one of 12 interviewers, each responsible for 4 clusters, who confirmed them by visiting women and their families at home and arranging to revisit for a postnatal interview at about 6 weeks after delivery. Interviewers had had higher secondary schooling, were trained for two weeks and met for feedback and ongoing training weekly. The interview was based on a predominantly closed questionnaire with questions on demography and socioeconomic factors, maternity history, antenatal, delivery, postnatal, and newborn care, illness and care seeking. The instrument was developed collectively over multiple iterations which involved piloting by interviewers, supervisors and project officers. In the event of a maternal, infant or child death, one of six supervisors visited to complete verbal autopsy.

After an explanation of the data collection activities, participants were asked for verbal consent to interview and assured of the confidentiality of data. Team members who encountered illness in mothers or infants had an ethical responsibility to recommend that they visit a health facility. Each completed interview was checked by the interviewer on site and by a supervisor. Supervisors also visited homes to crosscheck every tenth interview, and observed interviews randomly. Every third interview tool was checked by a project officer and supervisors and officers met weekly to review progress. Completed records of events and questionnaires were transferred to the central office weekly, and all were crosschecked. Two types of data were entered into electronic relational database management systems in Microsoft Access (Microsoft Corporation). Births and deaths were entered in a database dedicated to tracking follow-up and generating mortality rate outcomes. Interview questionnaires – where successfully completed – were entered in a separate database. Both databases included validation constraints and enforced referential integrity. The data management officer checked electronic data from every tenth questionnaire, and compared every fifteenth questionnaire entered against its original. Information provided by participants remained confidential. Access was restricted to interviewers, supervisors, data auditors, officers, and analysts. No analyses or outputs included the names of participants.

### Statistical analysis

The sample size was based on the later trial outcome of neonatal mortality. We aimed to accumulate 80–100 births per cluster per year, implying a cluster size of 900–1400 households on the basis of municipal demographic data [37]. We defined stillbirth as the death of an infant before birth, at a gestation greater than 22 weeks. Neonatal death was defined as the death of a live-born infant before 28 complete days. Results are presented as frequencies and percentages, both overall and for quartiles of socioeconomic status. We described socioeconomic status on the basis of a composite score generated for each participant, using standardised weights for the first component of a principal components analysis, a common approach recommended in the literature [38,39]. The final set of descriptive variables included house ownership, house construction, possession of a ration card, source of electricity, type of toilet and possession of a range of consumer durables. Socioeconomic scores were then ordered and divided by quartiles. We chose quartiles because they conveyed a simple and rapidly understandable message. The results did not differ in character when presented by either tertiles or quintiles.

We used random effects logistic regression to test the effect of socioeconomic quartile (as an ordered independent variable) on a range of outcomes as dependent binary variables. Quadrature checks supported the use of this approach to adjust for the clustered nature of the data. A similar analysis using individual socioeconomic scores did not alter the findings substantially. We compared the odds in the least poor quartile group with odds in the poorest in the same way, using an independent binary variable in which the least poor quartile took a value of 1 and the poorest a value of 0. Intervening quartiles were omitted from this analysis. We used random effects poisson regression for the comparison of stillbirth and neonatal mortality rates at cluster level, although a range of approaches yielded similar results. The results are presented as odds ratios or rate ratios with 95% confidence intervals. Socioeconomic differentials in home births and neonatal mortality were illustrated using concentration curves; the cumulative proportion of the outcome on the *y *axis was plotted against the cumulative proportion of births, ranked by socioeconomic quartile, on the *x *axis. For the neonatal mortality curve, we used outcome data for which we did not always have individual socioeconomic scores. We imputed a mean score to each cluster and divided the resulting 48 scores into quartile groups of 12 clusters. Concentration indices were computed from grouped data with recommended equations [40].

### Role of the funding source

The sponsors had no role in the study design, data collection, analysis, interpretation or writing of the article. DO had full access to all study data and final responsibility for the decision to submit for publication.

## Results

**Figure 1:**
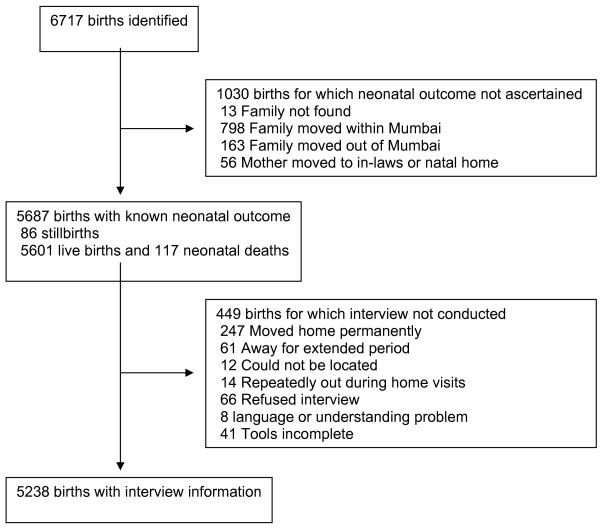
**Study profile**.

Figure 1 shows the study profile. We identified 6717 births over the year 1^st ^October 2005 to 30^th ^September 2006. The main reason for loss to ascertainment was family movement either within or out of Mumbai. We are certain of the neonatal outcome for 5687 of these (85%), and were able to complete interviews with 5238 women or their family members (78% of all births). Table 1 presents information on these women. Characteristics are summarised by socioeconomic quartile group, with odds ratios for the differences observed across groups, and a ratio for the proportion in the highest socioeconomic quartile group compared with the lowest. This is analogous to a 'rich:poor ratio', but we emphasise that the wealthiest are simply the least poor quartile group in a vulnerable urban slum. The less poor a woman's family, the less likely she was to have married and conceived in her teenage years (Odds Ratio 0.7, 95% Confidence Interval 0.7–0.8). Women in the highest group were twice as likely to have gone to school, and five times as likely to be able to read. The differences according to socioeconomic status represented by odds ratios were all significant at p < 0.001, apart from those seen for Hindu (not significant) and Muslim families (p = 0.01 for cross-quartile analysis and p = 0.23 for least poor: poor ratio).

**Table 1:** Characteristics, by cluster socioeconomic quartile group, of women who gave birth in urban slum communities under surveillance, Mumbai 2005–6

			Quartile group											
					
	All	(%)	1st	(%)	2nd	(%)	3rd	(%)	4th	(%)	OR	(95% CI)	Least poor: poorest	(95% CI)
	
Age at marriage														
Under 20	3635	(69)	1029	(79)	979	(75)	892	(68)	735	(56)	0.74	(0.69–0.79)	0.44	(0.35–0.55)
20 or over	1603	(31)	280	(21)	329	(25)	420	(32)	574	(44)				
Age at first pregnancy														
Under 20	2622	(50)	774	(59)	699	(53)	628	(48)	521	(40)	0.82	(0.78–0.87)	0.54	(0.44–0.65)
20 or over	2616	(50)	535	(41)	609	(47)	684	(52)	788	(60)				
Education														
No schooling	1477	(28)	605	(46)	437	(36)	264	(20)	141	(11)	0.56	(0.52–0.60)	0.15	(0.12–0.20)
Primary	367	(7)	125	(10)	92	(7)	93	(7)	57	(4)				
Secondary	3014	(58)	543	(41)	694	(53)	865	(66)	912	(70)				
College	380	(7)	36	(3)	55	(4)	90	(7)	199	(15)				
Literacy	3538	(67)	650	(50)	773	(59)	987	(75)	1128	(86)	1.74	(1.63–1.86)	5.56	(4.40–7.02)
Religion														
Hindu	2480	(47)	415	(32)	600	(46)	712	(54)	753	(57)	1.01	(0.94–1.09)	0.95	(0.73–1.23)
Muslim	2411	(46)	858	(65)	638	(49)	474	(36)	441	(34)	0.90	(0.83–0.98)	0.84	(0.64–1.11)
Other	347	(7)	36	(3)	70	(5)	126	(10)	115	(9)				
Family type														
Nuclear	2925	(56)	1020	(78)	864	(66)	578	(44)	463	(35)	0.48	(0.45–0.51)	0.11	(0.09–0.15)
Joint or extended	2313	(44)	289	(22)	444	(34)	734	(56)	846	(65)				
Duration of residence														
Less than a year	1427	(27)	448	(34)	382	(29)	317	(24)	280	(21)	0.75	(0.71–0.80)	0.49	(0.40–0.60)
1–10 years	2798	(54)	679	(52)	694	(53)	693	(53)	732	(56)				
More than 10 years	1013	(19)	182	(14)	232	(18)	302	(23)	297	(23)				
														
All	5238	(100)	1309	(100)	1308	(100)	1312	(100)	1309	(100)				

OR: odds ratio adjusted for clustering with random effects logistic regression, with socioeconomic quartile group as an ordered independent variable.

Least poor:poorest: odds ratio adjusted for clustering with random effects logistic regression, with a binary variable representing the 1^st ^and 4th socioeconomic quartiles as the independent variable.

Levels of home ownership (with at least notional tenure) were over 60% and most houses were of permanent construction. 3097 (59%) of families possessed ration cards, water supply and toilet facilities were generally shared, and 222 households (4%) had their own toilets. About a quarter of women had moved to their present residence in the last year, but 13% had been born there. Socioeconomic status was related to longevity and family structure. The poorest women were 10 times as likely to be living in nuclear families as the least poor, and were twice as likely to have moved to their current home in the preceding year.

Table 2 summarises reports of antenatal, postnatal and delivery care. Uptake of antenatal care was high. 86% of women made at least the recommended three visits, but the least poor group were almost five times as likely to have done so as the poorest (OR 4.9, 95% CI 3.5–6.9). There was a socioeconomic gradient towards more private sector care with increasing socioeconomic status, and also towards a greater likelihood of having obstetric ultrasonography (1.7, 1.5–1.9), tetanus toxoid (1.2, 1.0–1.4) and haematinic supplements (1.4, 1.3–1.5).

**Table 2:** Antenatal, postnatal and delivery care, by cluster socioeconomic quartile group, for women who gave birth in urban slum communities under surveillance, Mumbai 2005–6

			Quartile group											
					
	All	(%)	1st	(%)	2nd	(%)	3rd	(%)	4th	(%)	OR	(95% CI)	Least poor: poorest	(95% CI)
	
*Antenatal care*														
Had any antenatal care	4828	(92)	1135	(87)	1177	(90)	1249	(95)	1267	(97)	1.65	(1.47–1.85)	4.60	(3.05–6.94)
3 or more visits n = 4828	4531	(86)	1011	(77)	1081	(83)	1197	(91)	1242	(95)	1.72	(1.57–1.89)	4.94	(3.54–6.89)
Site of most antenatal care in Mumbai n = 4521														
Public sector	2075	(46)	609	(57)	565	(51)	518	(45)	382	(32)	0.75	(0.70–0.79)	0.38	(0.30–0.47)
Private sector	2447	(54)	453	(43)	540	(49)	643	(55)	811	(68)				
Had ultrasonography	4227	(88)	876	(77)	1006	(85)	1136	(91)	1209	(95)	1.69	(1.53–1.86)	5.00	(3.51–7.14)
Had tetanus toxoid	4632	(96)	1079	(95)	1121	(95)	1193	(95)	1239	(98)	1.19	(1.02–1.38)	2.10	(1.25–3.53)
Took iron tablets	4251	(81)	959	(73)	1016	(78)	1104	(84)	1172	(89)	1.40	(1.30–1.51)	3.00	(2.31–3.90)
														
*Postnatal care*														
Had a postnatal check	3438	(65.6)	740	(56.5)	819	(62.7)	894	(68.1)	985	(75.2)	1.26	(1.18–1.34)	2.13	(1.70–2.67)
Site of postnatal check in Mumbai n = 3101														
Public sector	1825	(59)	508	(76)	469	(63)	459	(57)	389	(44)	0.65	(0.60–0.70)	0.26	(0.20–0.34)
Private sector	1276	(41)	161	(24)	272	(37)	347	(43)	496	(56)				
														
*Delivery in Mumbai n = 4293*														
Home	480	(11)	262	(24)	141	(13)	49	(5)	28	(3)	0.54	(0.48–0.62)	0.17	(0.10–0.27)
Institutional														
Public sector	2335	(54)	606	(57)	619	(59)	626	(58)	484	(44)	0.66	(0.61–0.71)	0.27	(0.21–0.35)
Private sector	1478	(34)	200	(19)	293	(28)	397	(37)	588	(53)				
Caesarean section	565	(15)	100	(12)	117	(13)	152	(15)	196	(18)	1.18	(1.08–1.28)	1.58	(1.21–2.05)
Infant sex n = 5131														
Female	2406	(47)	601	(47)	683	(54)	667	(52)	688	(53)				
Male	2725	(53)	687	(53)	587	(46)	614	(48)	604	(47)	0.99	(0.94–1.04)	1.00	(0.85–1.16)
Low birth weight (<2500 g) n = 4343	1024	(24)	231	(26)	248	(24)	305	(26)	240	(19)	0.91	(0.85–0.97)	0.70	(0.57–0.87)
Infant had BCG immunization	4619	(90)	1054	(82)	1135	(89)	1204	(94)	1226	(95)	1.48	(1.33–1.64)	2.89	(2.02–4.14)
														
All	5238	(100)	1309	(100)	1308	(100)	1312	(100)	1309	(100)				

OR: odds ratio adjusted for clustering with random effects logistic regression, with socioeconomic quartile group as an ordered independent variable.

Least poor:poorest: odds ratio adjusted for clustering with random effects logistic regression, with a binary variable representing the 1^st ^and 4th socioeconomic quartiles as the independent variable.

4293/5238 (82%) women had their deliveries in Mumbai, and there was a significant but small increase in the likelihood of this with increasing socioeconomic quartile (OR 1.2; 95% CI 1.1–1.3). Of those who delivered in the city, 11% did so at home, 54% in public sector and 34% in private sector institutions. The operative delivery rate was 15% overall. Table 2 shows that these characteristics all had socioeconomic gradients. Women in the least poor group were less than one-fifth as likely to deliver at home as women in the poorest group (0.2, 0.1–0.3) and about a quarter as likely to deliver in the public sector (0.3, 0.2–0.4). The aggregate sex ratio was 882 females per 1000 males and there was no difference in sex ratio across socioeconomic groups. Of the 85% of infants for whom birth weight had been documented, 24% were low birth weight (<2500 g). Rising socioeconomic status was associated with a lower prevalence of low birth weight (0.9, 0.8–1.0). Immunisation rates for BCG were over 90%, but even at this level there was a significant tendency to higher rates in higher socioeconomic groups (1.5, 1.3–1.6). 63% of women had a postnatal check in the six weeks after delivery. Of those who had postnatal care in Mumbai, there was a trend towards private sector care with rising socioeconomic status, such that women in the least poor group were about four times less likely to use the public sector (0.3, 0.2–0.3). As with Table 1, the observed differences in Table 2 were all significant at p < 0.001, apart from those for antenatal receipt of tetanus toxoid (p = 0.03 for cross-quartile analysis and p = 0.005 for least poor: poor ratio) and for low birth weight (p = 0.004 for cross-quartile analysis).

**Figure 2:**
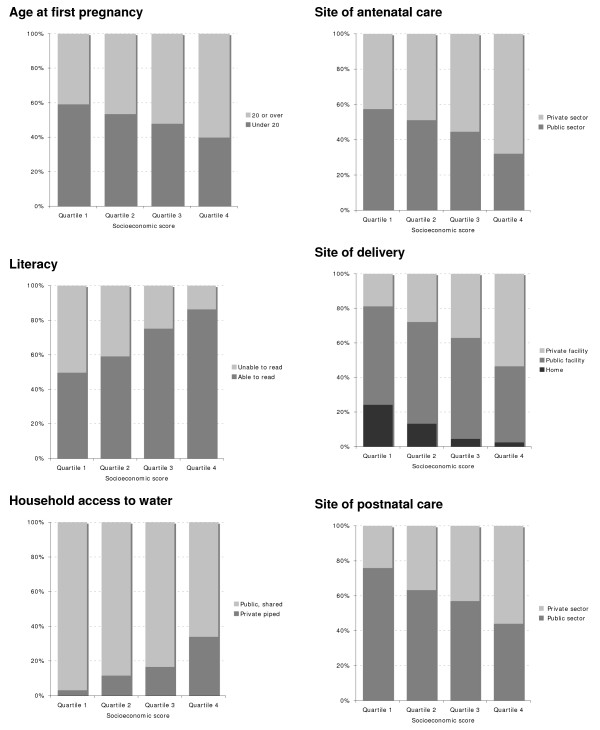
**Stacked bar charts showing differences in selected variables across socioeconomic quartile groups**.

**Figure 3:**
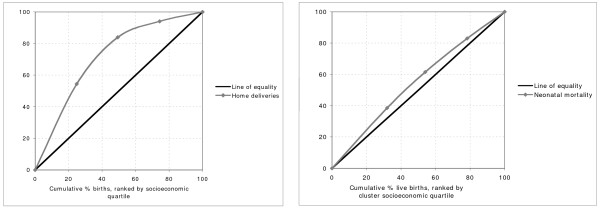
**Concentration curves for home delivery and neonatal mortality, by cluster socioeconomic quartile group**.

Table 3 summarises births, stillbirths and neonatal deaths by cluster socioeconomic quartile. Stillbirth rates did not vary with socioeconomic status. Neonatal mortality rates appeared to fall as socioeconomic status increased, although this did not attain significance. Figures 2 and 3 are included to reinforce visually the impression of generality and regularity in the socioeconomic differentials observed across a range of outcomes. Figure 2 shows six stacked bar charts, chosen to illustrate key respondent characteristics and the use of public and private sector care. Figure 3 shows concentration curves for a proximate outcome (home birth) and a distal outcome (neonatal mortality). A curve located above the line of equality suggests that the outcome in question occurs disproportionately in the poorer quantiles. Both curves suggest inequality of distribution across socioeconomic groups, the lower quartiles contributing greater proportions than the higher quartiles, but the inequality is more marked for home births than for neonatal mortality. Concentration indices support the impression given by the curves: -0.34 for home deliveries and -0.09 for neonatal deaths, implying a reduction in incidence as socioeconomic status rises.

**Table 3:** Births, stillbirths and neonatal deaths, by cluster socioeconomic quartile group, for women who gave birth in urban slum communities under surveillance, Mumbai 2005–6

		Quartile group							
						
	All	1st	2nd	3rd	4th	RR	(95% CI)	Least poor: poorest	(95% CI)
							
Births	5687	1816	1253	1391	1227				
Stillbirths	86	31	13	28	14				
Livebirths	5601	1785	1240	1363	1213				
Neonatal deaths	117	45	27	25	20				
									
Stillbirth rate per 1000 births	16.5	18.3	10.1	22.4	15.2	1.02	(0.74–1.40)	0.83	(0.28–2.44)
Neonatal mortality rate per 1000 live births	20.9	25.2	21.8	18.3	16.5	0.88	(0.71–1.08)	0.67	(0.32–1.39)

RR: rate ratio adjusted for clustering with random effects poisson regression, with socioeconomic quartile group as an ordered independent variable.

Least poor:poorest: rate ratio adjusted for clustering with random effects poisson regression, with a binary variable representing the 1^st ^and 4th socioeconomic quartiles as the independent variable.

## Discussion

We found inequalities between socioeconomic groups across a range of indicators. Poorer groups had less advantageous demographic and environmental profiles, a greater likelihood of seeking healthcare in the public sector, and indicators of compromised newborn health. Analyses of this type have usually been applied across the population spectrum from richest to poorest, or in rural areas [41], and we were struck by the regularly stepped picture of inequalities within the urban poor, a group that might inadvertently be considered relatively homogeneous.

Limits to the study included the sampling frame, cluster size, loss to follow-up, the omission of certain groups, and the methods used to assess poverty. The sampling frame included clusters selected randomly in six urban wards selected purposively as programme sites. Some wards were home to more slum residents than others, and we should be realistic about how likely they were to represent the experience in Mumbai slums as a whole, particularly since the sampling was stratified into eight clusters per ward. Added to this was the difficulty of ascertaining outcomes in urban communities with high levels of migration – about 27% per annum – and the occurrence of slum demolition and either in-situ or ex-situ rehabilitation. It is also possible that the study mortality rates were underestimates, because women who chose to deliver in rural homes, where mortality rates may be higher, were more likely to be lost to follow-up. Finally, birth surveillance was initiated in clusters of a minimum 1000 households, and we were aware from the outset that the system tended to exclude the very poorest residents of Mumbai, who are mobile, pavement-dwelling individuals or groups without fixed abode. We think, nevertheless, that our findings were robust. An observed crude birth rate of about 25 per 1000 in vulnerable areas suggests a high pick-up rate and compares with a national urban estimate of 19 per 1000 [42], and our aggregate findings on uptake of antenatal and postnatal care, institutional delivery and neonatal mortality rate agree with those of India's most recent National Family Health Survey (NFHS-3, 2005–6) [20,42].

There are limits to our classification of socioeconomic status. The criteria by which we defined vulnerability were necessarily diverse, and it is unclear how accurately they reflected real health risks; we intend to evaluate this in subsequent work. The main approaches to describing urban poverty are either economic (based on income, consumption or assets) or agreed through participatory work with communities [43]. India's poverty line assessments are based on income, but this has its weaknesses [44]. The asset-based and community-guided methods have in common that they tend to produce relative rather than absolute classifications, which is appropriate to the purpose of our analysis. Although this makes cross-site comparisons difficult, it does accord with an emphasis on inequalities.

The female-to-male sex ratio was very low. A recent aggregate urban estimate was 908 per 1000 in children under 6 [42]. One might expect a lower figure in the neonatal period, since early mortality is higher in male infants, but not as low as 882 per 1000. However, we did not show a decreasing sex ratio with increasing socioeconomic status (which has been seen in recent figures that span the socioeconomic spectrum) [45], and perhaps we should be more surprised that the overall figure for the poor families in our study is so low. This is worrying and certainly merits further study.

The clarity of the trend toward private sector care was striking. In this sense, slum residents are up-to-date: outpatient care in Asia is dominated by the private sector [46], and the figure for India is over 80% [47]. Countrywide, about 42% of institutional births are now in the public sector and 56% in the private sector [42]. In Mumbai, private sector health services fall into three categories: hospitals, nursing homes, and clinics. The Centre for Enquiry in Health and Allied Themes estimates that there are over 1000 private hospitals functioning in the city [48], and private practice is booming in the slums [33]. While the use of private services rises with socioeconomic status, a finding which agrees with earlier work [49], public sector maternity homes tend to be underutilized [29]. The perception is that private services are better than government services [50]. The effect of private care on household finances may be substantial. We know that urban women spend more on private practitioners and medicines than rural women [47]. Public expenditure on health care in India is among the lowest in the world, while it ranks in the top 20 for private spending. The burden of expense means that 40% of people admitted to hospital have to borrow money or sell assets [51], and it is thought that more than 20 million people fall below the poverty line each year as a result of out-of-pocket health care spending [52].

That slums are not homogeneous has been repeatedly pointed out [23,53,54]. Slums are a housing solution for the urban poor [55], and as home to more than half of Mumbai's people they must be diverse. The situation in a chawl (a tenement associated with an employer, classically the now defunct textile mills) bears little resemblance to the situation in a squatter settlement at the edge of the city. To a degree, our findings reflect longevity. Recent arrivals in an area tend to be poorer, less educated, have married and conceived younger and live with their partners in a nuclear family. With time, families expand, education increases, roots are put down, housing quality improves, and with these comes an aspiration for private health care. Space remains a constraint, however: 64% of homes had a television but only 4% a toilet.

## Conclusion

We can and should take a more discerning approach to urban health. All slums are not the same, and each is home to a variety of people who face different exposures and may respond differently to risk. Nevertheless, we are still aware of a tendency to see people who live in slums as homogeneously disadvantaged. We can confirm the assumption that aggregate data mask significant intra-urban differences, but the clarity with which a pattern emerged surprised us. The poorest slum residents are more dependent upon public sector health care, and there is a risk of the system becoming two-tier. Simultaneously, our demonstration of a regular progression towards the private sector raises questions about its quality and regulation. If this progression is, as it seems, inexorable, we need to begin to conceive health systems in terms of chaotic composites of (informal and formal) private and public providers.

## Competing interests

The authors declare that they have no competing interests. DO had full access to all the data in the study and had final responsibility for the decision to submit for publication.

## Authors' contributions

All authors contributed to critique and modification, and read and approved the final manuscript. NSM was the project coordinator, contributed to the design of the study, and participated in data analysis and interpretation. UB and SD contributed to the study design, were responsible for management of data collection and entry, and participated in data analysis and interpretation. SB was research advisor to the study and contributed to its design. AC is the director of the UCL Centre for International Health and Development, contributed to the design of the study, and had overall responsibility for UK partner contributions. AF is the director of SNEHA, contributed to the design of the study, and had overall responsibility for Mumbai work. DO contributed to the study design, data management, analysis and interpretation, and wrote the first draft of the article.

## References

[B1] United Nations (2004). World urbanization prospects: the 2003 revision.

[B2] Vlahov D, Freudenberg N, Proietti F, Ompad D, Quinn A, Nandi V, Galea S (2007). Urban as a determinant of health. J Urban Health: Bull New York Acad Med.

[B3] Ooi G, Phua K (2007). Urbanization and slum formation. J Urban Health.

[B4] United Nations Human Settlements Programme (UN-Habitat) (2003). The challenge of slums: global report on human settlements 2003.

[B5] Caldwell J, Caldwell B (2002). Poverty and mortality in the context of economic growth and urbanization. Asia-Pacific Popul J.

[B6] Satterthwaite D (2004). The scale of urban change worldwide 1950–2000 and its underpinnings.

[B7] Bhargava S, Singh K, Saxena B (1991). ICMR Task Force National Collaborative Study on identification of high risk families, mothers and outcome of their off-springs with particular reference to the problem of maternal nutrition, low birth weight, perinatal and infant morbidity and mortality in rural and urban slum communities. Indian Pediatr.

[B8] Awasthi S, Pande V, Glick H (1996). Under fives mortality in the urban slums of Lucknow. Ind J Pediatr.

[B9] Kapoor R, Srivastava A, Mishra P, Sharma B, Thakur S, Srivastava K, Singh G (1996). Perinatal mortality in urban slums in Lucknow. Indian Pediatr.

[B10] Hussain A, Ali S, Kvale G (1999). Determinants of mortality among children in the urban slums of Dhaka city, Bangladesh. Trop Med Int Health.

[B11] D'Souza R, Bryant J (1999). Determinants of childhood mortality in slums of Karachi, Pakistan. J Health Popul Dev Ctries.

[B12] Bhandari N, Bahl R, Taneja S, Martines J, Bhan M (2002). Pathways to infant mortality in urban slums of Delhi, India: implications for improving the quality of community- and hospital-based programmes. J Health Popul Nutr.

[B13] Fernandez A, Mondkar J, Mathai S (2003). Urban slum-specific issues in neonatal survival. Indian Pediatr.

[B14] Awasthi S, Agarwal S (2003). Determinants of childhood mortality and morbidity in urban slums in India. Indian Pediatr.

[B15] Stephens C, Harpham T, Chaane B (1991). Slum improvement: health improvement? PHP Departmental Publication No 1.

[B16] Mutatkar R (1995). Public health problems of urbanization. Soc Sci Med.

[B17] Sclar E, Garau P, Carolini G (2005). The 21st century health challenge of slums and cities. Lancet.

[B18] Ingle G, Nath A (2006). Reaching out to the unreached: health care for the poor in India. Indian J Commun Med.

[B19] NNF (2004). The State of India's Newborns.

[B20] Government of India Ministry of Health and Family Welfare (2007). 2005–2006 National Family Health Survey (NFHS-3). Fact sheet: Maharashtra (provisional data).

[B21] Harpham T, Lusty T, Vaughan P (1988). In the shadow of the city. Community health and the urban poor.

[B22] Timaeus I, Lush L (1995). Intra-urban differentials in child health. Health Transition Review.

[B23] Kausar F, Griffiths P, Matthews Z (1999). Poverty and maternal health care utilisation in Maharashtra: associated influences on infant mortality and morbidity. Working Paper No 20.

[B24] Unger A, Riley L (2007). Slum health: from understanding to action. PLoS Med.

[B25] de Zoysa I, Bhandari N, Akhtari N, Bhan M (1998). Careseeking for illness in young infants in an urban slum in India. Soc Sci Med.

[B26] Gupta H, Baghel A (1999). Infant mortality in the Indian slums: case studies of Calcutta metropolis and Raipur city. Int J Population Geography.

[B27] Vaid A, Mammen A, Primrose B, Kang G (2007). Infant mortality in an urban slum. Indian J Pediatr.

[B28] Fikree F, Ali T, Durocher J, Rahbar M (2005). Newborn care practices in low socioeconomic settlements of Karachi, Pakistan. Soc Sci Med.

[B29] Matthews Z, Brookes M, Stones R, Hossein B, Moore S (2005). Village in the city: autonomy and maternal health-seeking among slum populations of Mumbai. A focus on gender: collected papers on gender using DHS data.

[B30] Census of India (2001). http://www.censusindia.gov.in/.

[B31] Fernandez A, Osrin D (2006). The City Initiative for Newborn Health. PLoS Med.

[B32] Verma M (2006). Recommended policy guidelines for public health. A report to the Municipal Corporation of Greater Mumbai and the Non-Governmental Organization Council. Mumbai.

[B33] Garner P, Thaver I (1993). Urban slums and primary health care. The private doctor's role. BMJ.

[B34] Shah More N, Bapat U, Das S, Patil S, Porel M, Vaidya L, Koriya B, Barnett S, Costello A, Fernandez A, Osrin D (2008). Cluster-randomised controlled trial of community mobilisation in Mumbai slums to improve care during pregnancy, delivery, postpartum and for the newborn. Trials.

[B35] Osrin D, Manandhar A, Shrestha A, Mesko N, Tumbahangphe K, Shrestha D, Manandhar D, Costello A (2003). Design of a surveillance system for pregnancy and its outcomes in rural Nepal. J Nepal Med Assoc.

[B36] Barnett S, Nair N, Tripathy P, Borghi J, Rath S, Costello A (2008). A prospective key informant surveillance system to measure maternal mortality – findings from indigenous populations in Jharkhand and Orissa. BMC Pregnancy Childbirth.

[B37] Hayes R, Bennett S (1999). Simple sample size calculation for cluster-randomized trials. Int J Epidemiol.

[B38] Filmer D, Pritchett L (2001). Estimating wealth effects without expenditure data – or tears: an application to educational enrollments in states of India. Demography.

[B39] Vyas S, Kumaranayake L (2006). Constructing socio-economic status indices: how to use principal components analysis. Health Policy Plan.

[B40] Kakwani N, Wagstaff A, van Doorslaer E (1997). Socioeconomic inequalities in health: measurement, computation and statistical inference. J Econometrics.

[B41] Armstrong Schellenberg J, Victora C, Mushi A, de Savigny D, Schellenberg D, Mshinda H, Bryce J, for the Tanzania IMCI MCE baseline household survey study group (2003). Inequities among the very poor: health care for children in rural southern Tanzania. Lancet.

[B42] Government of India Ministry of Health and Family Welfare (2007). National Family Health Survey, India (NFHS-3 2005-06).

[B43] Wratten E (1995). Conceptualizing urban poverty. Environment & Urbanization.

[B44] Swaminathan M, Patel S, Masselos J (2003). Aspects of poverty and living standards. Bombay and Mumbai: the city in transition.

[B45] Guilmoto C (2007). Characteristics of sex-ratio imbalance in India, and future scenarios. Hyderabad: 4th Asia Pacific Conference on Reproductive and Sexual Health and Rights.

[B46] Aljunid S (1995). The role of private medical practitioners and their interactions with public health services in Asian countries. Health Policy Plan.

[B47] Bhatia J, Cleland J (2001). Health-care seeking and expenditure by young Indian mothers in the public and private sectors. Health Policy Plan.

[B48] Madhiwalla N, Patel S, Masselos J (2003). Hospitals and city health. Bombay and Mumbai: the city in transition.

[B49] Yesudian C (1994). Behaviour of the private sector in the health market of Bombay. Health Policy Plan.

[B50] Griffiths P, Stephenson R (1999). Prenatal care and child delivery in Maharashtra: a qualitative approach. Working Paper no. 1999–01.

[B51] Sengupta A, Nundy S (2005). The private health sector in India. Br Med J.

[B52] National Sample Survey Organisation National Sample Survey, 52nd Round, 1995–1996.

[B53] Yesudian C (1988). Health service utilisation in urban India.

[B54] O'Hare G, Abbot D, Barke M (1998). A review of slum housing policies in Mumbai. Cities.

[B55] Burra S (2005). Towards a pro-poor framework for slum upgrading in Mumbai, India. Environment & Urbanization.

